# Spatial Variation of *Cladophora* Epiphytes in the Nan River, Thailand

**DOI:** 10.3390/plants10112266

**Published:** 2021-10-22

**Authors:** Karnjana Ruen-Pham, Linda E. Graham, Anchittha Satjarak

**Affiliations:** 1 Plants of Thailand Research Unit, Department of Botany, Faculty of Science, Chulalongkorn University, Bangkok 10330, Thailand; k.ruenpham@gmail.com; 2 Department of Botany, University of Wisconsin-Madison, 430 Lincoln Drive, Madison, WI 53706, USA; lkgraham@wisc.edu

**Keywords:** *Cladophora*, microbiome, Nan River, lotic environment, ecological services

## Abstract

*Cladophora* is an algal genus known to be ecologically important. It provides habitats for microorganisms known to provide ecological services such as biosynthesis of cobalamin (vitamin B_12_) and nutrient cycling. Most knowledge of microbiomes was obtained from studies of lacustrine *Cladophora* species. However, whether lotic freshwater *Cladophora* microbiomes are as complex as the lentic ones or provide similar ecological services is not known. To illuminate these issues, we used amplicons of 16S rDNA, 18S rDNA, and ITS to investigate the taxonomy and diversity of the microorganisms associated with replicate *Cladophora* samples from three sites along the Nan River, Thailand. Results showed that the diversity of prokaryotic and eukaryotic members of *Cladophora* microbiomes collected from different sampling sites was statistically different. Fifty percent of the identifiable taxa were shared across sampling sites: these included organisms belonging to different trophic levels, decomposers, and heterotrophic bacteria. These heterogeneous assemblages of bacteria, by functional inference, have the potential to perform various ecological functions, i.e., cellulose degradation, cobalamin biosynthesis, fermentative hydrogen production, ammonium oxidation, amino acid fermentation, dissimilatory reduction of nitrate to ammonium, nitrite reduction, nitrate reduction, sulfur reduction, polyphosphate accumulation, denitrifying phosphorus-accumulation, and degradation of aromatic compounds. Results suggested that river populations of *Cladophora* provide ecologically important habitat for microorganisms that are key to nutrient cycling in lotic ecosystems.

## 1. Introduction

*Cladophora* (Kützing) Kützing is a cosmopolitan filamentous green algal genus widely distributed in freshwater, marine, and brackish shoreline environments across arctic and tropical regions [1,2,3,4]. Freshwater species occur as filaments attached to various kinds of substrates, from rock surface to shells of arthropods, and as free-floating algal masses. This alga survives in water bodies of a wide range of nutrients levels, from the ultra-oligotrophic to the highly eutrophic, and a wide variety of conditions of attachment substrate, light intensity, water temperature, and water velocity [5].

Having unique characteristics and a high surface area, *Cladophora* is known as an ecological engineer that provides habitats for several other organisms, e.g., bacteria, archaea, protists, microalgae, fungi, and invertebrates, promoting complex epiphytic communities [6,7,8,9,10]. Previous studies found that these heterogeneous assemblages of living organisms displayed various ecological interactions, e.g., [4,6,7,9,10,11]. Predation in the algal microbiome could be inferred from the presence of organisms belonging to different trophic levels in the food chain, e.g., producers, herbivores, carnivores, and omnivores, representing organisms of diverse supergroups, e.g., Viridiplantae, Euglenozoa, Stramenopiles, Alveolata, Rhizaria, Amoebozoa, and Eumetazoa [12]. Competition could be inferred from the alternations or shifts of taxonomic richness and abundance in comparative studies e.g., [11]. For example, dynamic changes in bacterial populations were observed during the algal growth season [6] or when *Cladophora* grew in media supplemented with different chemical compositions [11], as it happens spontaneously also in the wild plant populations of terrestrial environments, where the same taxon to a different chemical soil composition may produce a different chemotype [13].

Among these ecological interactions, mutualism in the *Cladophora* microbiomes has been the most well studied, e.g., [4,6,7,9,10,11]. The mutualistic relationship was inferred from the presence of taxonomic markers and protein-coding genes in metagenomic analyses. Studies suggested that the photoautotrophs, i.e., the host *Cladophora* and epiphytic diatoms, provide habitats and exopolysaccharides that serve as resources for other heterotrophic bacteria and fungi. In return, the heterotrophic bacteria and fungi supply the phycosphere with vitamin B_12_, inorganic minerals, and gases needed for the growth of *Cladophora* and other microbes in the association [4,6,9,10,11].

Algal structural features, wide distribution, and ecological importance made the *Cladophora* microbiome a popular research topic during the past decade. Microbiome studies of the freshwater lacustrine *Cladophora* were conducted for algal samples collected from many locales, e.g., [4,6,9,10,11,14,15,16,17,18,19,20]. However, the microbiomes of freshwater *Cladophora* living in lotic water bodies have not been investigated.

In lotic environments, the directional flow of water makes nutrient cycling distinctively different from that of lentic environments. In temperate lakes, nutrients influx, e.g., debris, dissolved organic matter, and inorganic minerals, is mainly from seasonal turnover and water turbulence caused by wind, whereas the influx of nutrients in streams and rivers appears as pulses acquired from the adjacent floodplain normally caused by seasonal rainfall. The outflow of these available organic and inorganic compounds is also different. In lakes, losses are mainly by sedimentation, which makes these resources readily recyclable in a suitable condition. On the other hand, losses in the lotic systems seem to be less recyclable due to the unidirectional flow of water volume [21,22,23,24].

In the northern part of Thailand, growths of *Cladophora* regularly occur along river shorelines. Conspicuous populations in the Nan River, which is one of the main rivers of the northern part of Thailand, are harvested for culinary (“Kai”) and medicinal purposes [25,26,27,28,29,30,31] and making bioethanol [32]. Even so, the algal microbiome has never been investigated. Therefore, in this study, we used amplicon analyses to study the diversity of microbiota living in *Cladophora* sp. present along the Nan River. Results showed that the diversity of prokaryotes and eukaryotes in the algal microbiomes significantly differed among study sites. About half of the identifiable taxa were commonly present in all the replicates. These organisms, which include heterotrophic bacteria and decomposers, span multiple trophic levels, which suggests the presence of a complex food chain within the algal microbiome. Additionally, by functional inference, these organisms putatively provide various ecological functions in the Nan River.

## 2. Materials and Methods

**Figure 1:**
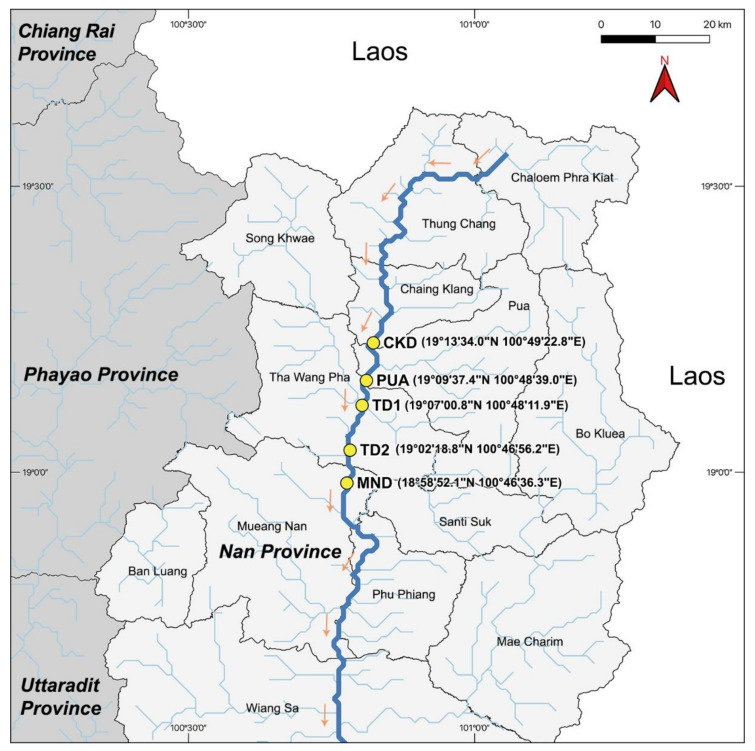
Five sample collecting sites in Nan River, Nan Province, located in four different districts. CKD located in Chiang Klang district, PUA located in Pua district, TD1 and TD2 located in Tha Wang Pha district, and MND located in Mueang Nan district. Only three locations (CKD, PUA, and TD1) were *Cladophora* dominant. Yellow dot represents each collecting site. Bold blue line represents the Nan River. Orange arrows represent the flow direction. Other blue lines represent other smaller rivers and streams.

Algal samples were collected from Nan River, Nan, Thailand, during the late growing season in March 2020. Five collecting sites with four replicates per site were located in four different districts—Chiang Klang (19°13′34.0” N 100°49′22.8” E: defined as CKD), Pua (19°09′37.4” N 100°48′39.0” E: defined as PUA), Tha Wang Pha (19°07′00.8” N 100°48′11.9” E and 19°02′18.8” N 100°46′56.2” E: defined as TD1 and TD2, respectively), and Mueang Nan District (18°58′52.1” N 100°46′36.3” E: defined as MND) as shown in Figure 1. The collected algal samples were submerged in DNA/RNA Shield^TM^ (Zymo Research, Irvine, CA, USA) and stored in sterile microcentrifuge tubes and Whirl-Pak^®^ (Nasco, Madison, WI, USA) before being brought back to the laboratory for the following analysis. Additionally, we obtained the secondary information of the water physical properties from the annual report available at Regional Environmental Office 2 Lampang (http://www.reo02.mnre.go.th/th/index; accessed on 27 August 2021). The information included values of the water quality index, dissolved oxygen, biological oxygen demand, and ammonia nitrogen content.

## 3. Algal Identification

At the laboratory, the algal samples were put into sterile petri dishes submerged in DNA/RNA Shield^TM^. Algal filaments were selected using sterile forceps and washed three times to remove loosely associated materials. Then, the alga samples were observed using an ECLIPSE E100 microscope (Nikon Corporation, Tokyo, Japan). Only samples from sites dominated by *Cladophora* were employed in this study. To select the *Cladophora* dominant samples, we used morphological characteristics of algal samples to identify the alga to the genus level [33]. To identify the dominant *Cladophora* species of each replicate, we used the assembled 18S rDNA amplicons to perform BLASTN search against the NCBI non-redundant nucleotide database (accessed on 13 September 2021).

## 4. Taxonomic Identification of Microbial Taxa

### Light Microscopy

When possible, microbes associated with filaments of the *Cladophora* host were identified to genus level using group-specific identification keys [34,35,36,37]. The permanent slides of algal specimens, included their replicates, have been deposited as barcode number BCU5004 (CKD), BCU5005 (PUA), and BCU5006 at Kasin Suvatabandhu Herbarium, Department of Botany, Chulalongkorn University, Thailand (https://www.chula.ac.th/museum/763/).

## 5. Taxonomic Informatic Analysis of Amplicons (SSU and ITs)

### 5.1. DNA Extraction and Sequencing

The *Cladophora* samples were washed three times using DNA/RNA Shield^TM^ to remove the dirt and debris. Then, genomic DNA was extracted using Quick-DNA^TM^ Fecal/Soil Microbe Kits (Zymo Research, Irvine, CA, USA). The 16S rRNA region V3-V4 was amplified using primers 341F and 805R [38], the 18S rRNA region V4 was amplified using primers Reuk454FWD1 and V4r [39], and the ITS was amplified using primers ITS-1F [40] and ITS-2R [41], as provided in Table S1. The amplicons were then purified, indexed, and sequenced using an Illumina MiSeq at Omics Sciences and Bioinformatics Center (Chulalongkorn University, Bangkok, Thailand).

### 5.2. Data Analysis

The quality of the 250 bp paired-end raw reads was assessed by FastQC [42]. Low-quality nucleotide bases were removed using Trimmomatic v. 0.39 using SLIDINGWINDOW:4:30 [43]. The taxa and the taxonomic diversity of the epiphytic microbiota of *Cladophora* sp. were identified and assessed by comparing the annotated rDNA against the SILVA ribosomal RNA gene database release 138.1 [44] using SILVAngs pipeline. In the workflow, trimmed reads were aligned using SILVA INcremental Aligner (SINA). Reads shorter than 50 aligned nucleotides, with ambiguities larger than 2%, or with homopolymers larger than 2% were eliminated before entering the downstream processes. Then, putative contaminant reads, including PCR artifacts and low-quality aligned reads (with alignment identity less than 50% and alignment score less than 40), were filtered out.

Reads that passed the filtered steps were then de-replicated. Identical reads were identified and clustered into different Operational Taxonomic Units (OTUs) using cd-hit-est [45] running in accurate mode, overlooking overhangs, and applying identity criteria of 1.00 and 0.98, respectively. Then for each cluster, the representative sequence was then classified by a local nucleotide BLAST search against the non-redundancy of the SILVA SSU Ref database release 138.1 using BLASTN with standard settings. The classifiable reads were mapped onto all reads that were assigned to the respective OTU. Lastly, to filter out chimeric reads, we searched the classified results against SILVA release 138.1 SSU using VSEARCH v. 2.8.3.0 [46] implemented in Galaxy v. 1.39.5.1 [47]. Then the taxonomic classification was called if the sequence returned BLASTN with a score of “(percentage sequence identity + percentage alignment coverage)/2” greater than or equal to 93. Otherwise, the OTUs were called “No relative”.

In addition to the SILVAngs pipeline, for detecting fungi, we used a local BLASTN by searching our assembled ITS amplicons against the UNITE database v. 7.2 [48] (sh_refs_qiime_ver7_97_01.12.2017.fasta available at https://dx.doi.org/10.15156/BIO/587481 (accessed on 16 November 2020) using E-value = 1 × 10^−10^. Then, we filtered out the chimeric reads by using VSEARCH v. 2.8.3.0 [46] implemented in Galaxy v. 1.39.5.1 by searching the obtained reads against the UCHIME reference datasets v. 7.1 [49] (uchime_reference_dataset_01.01.2016.fasta available at https://unite.ut.ee/sh_files/uchime_reference_dataset_01.12.2016.zip (accessed on 19 November 2020).

The raw data for the *Cladophora* microbiomes have been deposited in NCBI SRA BioProject PRJNA761577 and BioSample SAMN21356006 (CKD), SAMN21356007 (PUA), and SAMN21356008 (TD1).

## 6. Diversity Estimation

### 6.1. Alpha Diversity

The taxonomic abundance of the microbial taxa was assessed using the results returned from the SILVAngs pipeline. To assess the alpha-diversity, we firstly calculated the values of Shannon index, Simpson index, Chao1, and richness using functions namely diversity and estimateR implemented in the vegan package v. 2.5-7 [50] in RStudio v. 1.4.1106 [51]. To evaluate whether the values obtained from calculations were statistically different, values of One-way ANOVA (*p* < 0.05) and Tukey’s HSD were calculated using the agricolae package v. 1.3-5 [52]. Then, results were visualized using box plots implemented in the ggplot2 package v. 3.3.5 [53] in RStudio.

### 6.2. Beta Diversity

Beta-diversity index, Bray-Curtis dissimilarity, was calculated using the vegdist function in the vegan package [50] in RStudio. Then, to examine whether the values of Bray-Curtis dissimilarity were statistically different, we performed Analysis of Similarity (ANOSIM; function anosim, vegan package) and permutational Multivariate Analysis of Variance (perMANOVA; function adonis, vegan package) using 999 permutations. Results were then visualized using the Principal Coordinate Analysis (PCoA) with the cmdscale function.

## 7. Common Organisms in Lotic and Lentic Freshwater *Cladophora* Microbiome

To investigate the presence of taxa that are commonly present in freshwater *Cladophora* microbiomes, we re-analyzed the taxonomic classification using the SILVAngs pipeline described above for sequence read archive of metagenomic data from *Cladophora* collected from Lake Mendota (Madison, WI, USA) [6,9] and Lake Michigan (Portage, IN, USA) [7]. Then, we searched for the taxa commonly present in the lotic and lentic *Cladophora* microbiomes.

## 8. Results

### Identification of Cladophora sp.

**Figure 2:**
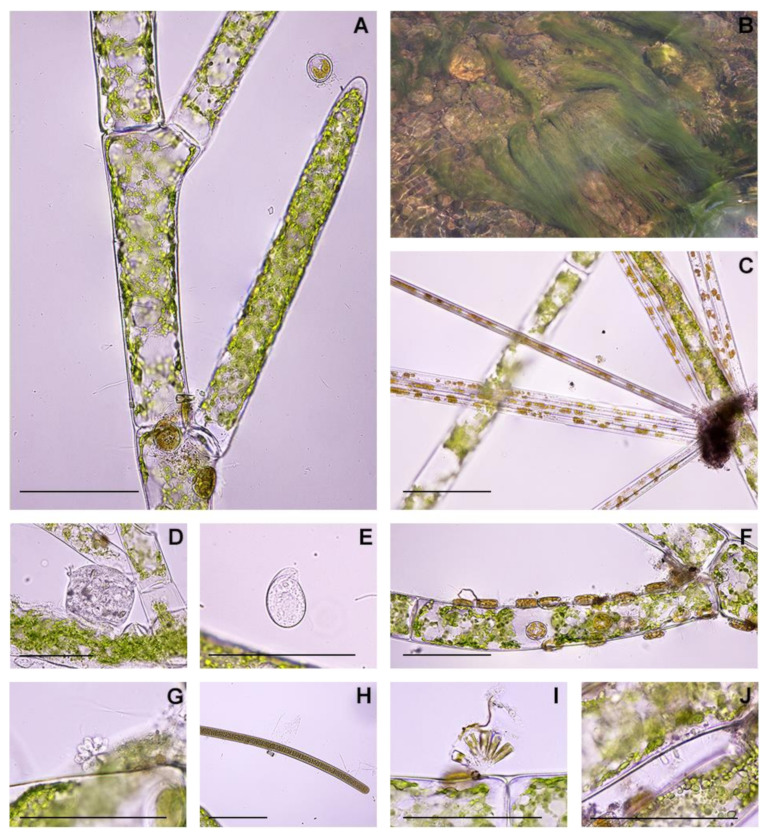
Morphological characteristic of *Cladophora* sp. collected from Nan River, Thailand, and epiphytic microbes under light microscopy. (**A**)—Morphology of *Cladophora* sp., showing branched monosiphonous filament with reticulate chloroplasts. (**B**)—Macroscopic *Cladophora* sp. filaments attached to substrate. (**C**)—Stramenopile *Synedra*. (**D**)—Ciliate *Vorticella*. (**E**)—other Ciliate. (**F**)—Stramenopile *Cocconeis*. (**G**)—Stramenopile *Synura*. (**H**)—Cyanobacterium *Oscillatoria*. (**I**)—Stramenopile *Gomphonema*. (**J**)—Cyanobacterium *Chamaesiphon*. Black scale bars: 100 μm.

Only the algal samples collected from three sites (CKD, PUA, and TD1) were morphologically identified to genus *Cladophora*, having distinct characteristics of monosiphonous and branched filaments with a unique branching style, where the lateral branch arisen under the septum between two cells of the main axis. Each cell in the filament contained reticulate chloroplasts and thick cell walls (Figure 2A). The algal basal region was attached to the substrates using holdfast. The filaments appeared as tufts, forming the macroscopic green algal bloom floating in water (Figure 2B).

In addition to having similar morphology, the *Cladophora* samples collected from the three sites also have identical amplicon sequences (Figure S1). BLASTN results suggested that the sequence was equally similar to published and unpublished freshwater *Cladophora* spp. reported from authors from several countries (Table S2).

## 9. Taxonomic Identification of Microbial Taxa

### 9.1. Light Microscopy

When observed under the light microscope, algal filaments were observed to host a high abundance of the photosynthetic stramenopile (diatom) *Cocconeis* C.G. Ehrenberg (Figure 2F) and the cyanobacterium *Chamaesiphon* A. Braun and Grunow (Figure 2J). In addition, photosynthetic stramenopiles *Synura* C.G. Ehrenberg, *Synedra* C.G. Ehrenberg*,* and *Gomphonema* C.G. Ehrenberg (Figure 2C,G,I), the *Vorticella* C. Linnaeus and other ciliates (Figure 2D,E), and cyanobacterium *Oscillatoria* Vaucher ex Gomont (Figure 2H) were always present in association with the alga.

### 9.2. Amplicon Analysis

#### 9.2.1. SSU—16S rDNA Amplicon Analysis

**Figure 3:**
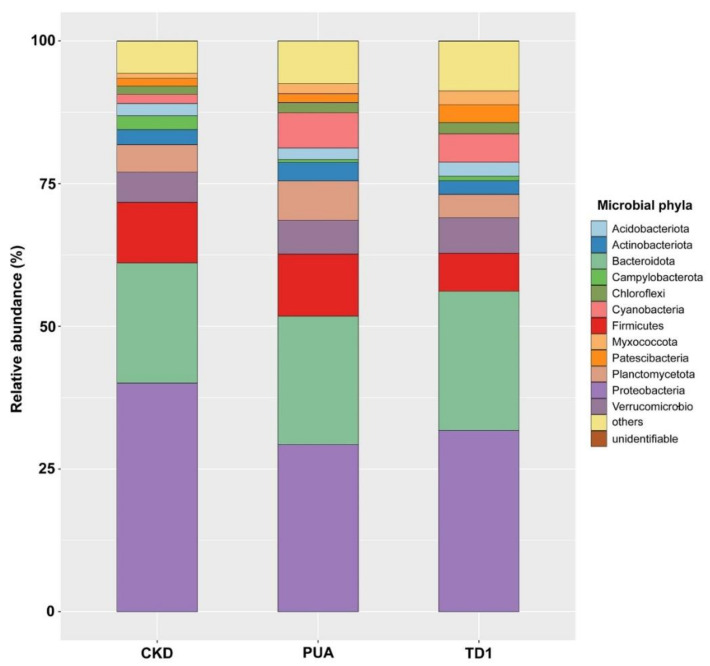
Results from 16S rDNA amplicon analysis of *Cladophora* sp. collected from Nan River, sites CKD, PUA, and TD1, showed that bacterial phyla and their relative abundance varied. The five most abundant phyla in site CKD were Proteobacteria (40.04%), Bacteroidetes (21.07%), Firmicutes (10.59%), Verrucomicrobia (5.30%), and Planctomycetes (4.80%). The five most abundant phyla in site PUA were Proteobacteria (29.29%), Bacteroidetes (22.50%), Firmicutes (10.86%), Planctomycetes (6.88%), and Cyanobacteria (6.20%). The five most abundant phyla in site TD1 were Proteobacteria (31.77%), Bacteroidetes (24.37%), Firmicutes (6.67%), Verrucomicrobia (6.22%), and Cyanobacteria (4.98%).

Amplicon analysis showed that bacterial phyla and their relative abundance varied (Figure 3). The five most abundant phyla in site CKD were Proteobacteria (40.04%), Bacteroidetes (21.07%), Firmicutes (10.59%), Verrucomicrobia (5.30%), and Planctomycetes (4.80%). The five most abundant phyla in site PUA were Proteobacteria (29.29%), Bacteroidetes (22.50%), Firmicutes (10.86%), Planctomycetes (6.88%), and Cyanobacteria (6.20%). The five most abundant phyla in site TD1 were Proteobacteria (31.77%), Bacteroidetes (24.37%), Firmicutes (6.67%), Verrucomicrobia (6.22%), and Cyanobacteria (4.98%). Bacterial taxa were classified into 689 distinct genera; 227 bacterial genera were present in all four replicates of the three collecting sites (Table S3).

The three collected sites in this study were located in the upper region of the Nan River, where the water flowed from CKD to PUA and TD1, respectively. We initially sampled the water from each site for physical and chemical measurements. However, all the assessment facilities were shut down for a few months, which made it not possible to obtain such parameters from the collecting sites. However, we compared the abundance of the common bacterial genera and found that some bacterial genera occurred as a gradient from the upper stream (CKD) to the lower stream of the river (TD1). Genera that had higher relative abundance in the upper stream included Proteobacteria *Acinetobacter, Aeromonas,* and *Vogesella,* Epsilonproteobacteria *Pseudarcobacter,* Firmicutes *Clostridium Sensu stricto 12*, and Bacteroidetes *Bacteroides* and *Flavobacterium.* In contrast, genera that had higher relative abundance in the lower stream included Bacteroidetes *Paludibacter*, Proteobacteria *Hydrogenophaga* and *Leptothrix*, and Firmicutes *Fusibacter* (Table S3).

#### 9.2.2. SSU—18S rDNA Amplicon Analysis

**Figure 4:**
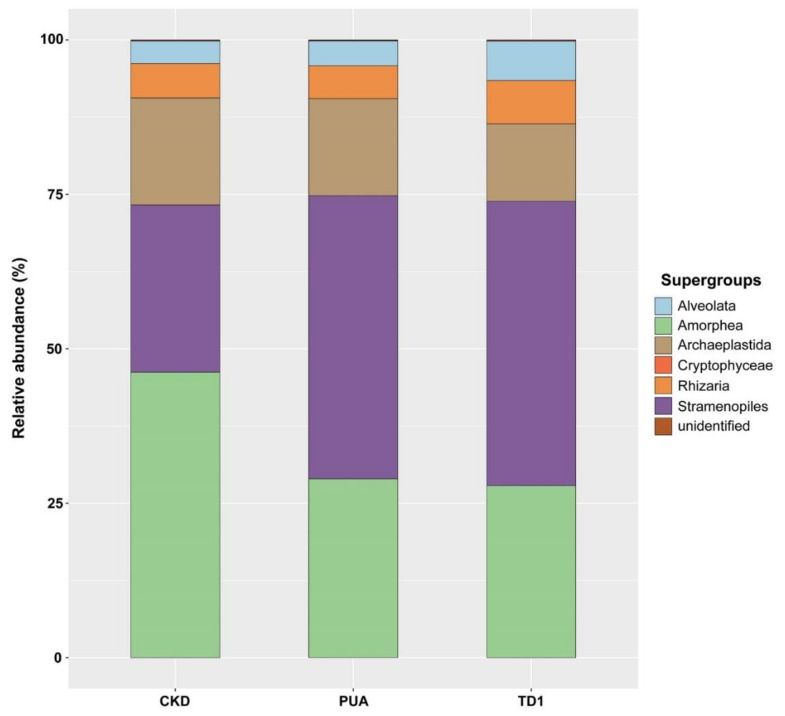
Results from 18S rDNA amplicon analysis of *Cladophora* sp. collected from Nan River, sites CKD, PUA, and TD1, showed that supergroups and their relative abundance were varied. The five supergroups with the highest relative abundance at site CKD were Amorphea (46.19%), Stramenopiles (27.10%), Archaeplastida (17.33%), Rhizaria (5.55%), and Alveolata (3.66%). Supergroups for site PUA were Stramenopiles (45.84%), Amorphea (28.95%), Archaeplastida (15.71%), Rhizaria (5.33%), and Alveolata (4.01%). Supergroups for site TD1 were Stramenopiles (46.04%), Amorphea (27.83%), Archaeplastida (12.55%), Rhizaria (7.02%), and Alveolata (6.37%).

The supergroups and their relative abundance obtained from 18S rDNA amplicon analysis were also varied. These included 575 genera belonging to Alveolata, Amorphea, Archaeplastida, Cryptophyceae, Rhizaria, and Stramenopiles (Table S3). The five supergroups with the highest relative abundance at site CKD were Amorphea (46.19%), Stramenopiles (27.10%), Archaeplastida (17.33%), Rhizaria (5.55%), and Alveolata (3.66%). Supergroups for site PUA were Stramenopiles (45.84%), Amorphea (28.95%), Archaeplastida (15.71%), Rhizaria (5.33%), and Alveolata (4.01%). Supergroups for site TD1 were Stramenopiles (46.04%), Amorphea (27.83%), Archaeplastida (12.55%), Rhizaria (7.02%), and Alveolata (6.37%) (Figure 4).

Among the annotated genera, 51 were present in all sites and some genera exhibited a higher relative abundance. These included Alveolata *Trochilia*, Amorphea *Nuclearia*, *Paramicrosporidium*, and *Sorodiplophrys*, Archaeplastida *Jaoa*, *Planktosphaeria*, *Spirogyra*, *Rhizoclonium*, Rhizaria *Heteromita*, and Stramenopiles *Aphanomyces*, *Cocconeis*, *Gomphonema*, *Leptolegnia*, *Ochromonas*, *Paraphysomonas*, *Poteriospumella*, and *Pythium*. In addition, genera that were more abundant in the upper stream included Amorphea *Paramicrosporidium,* Archaeplastida *Planktosphaeria,* and Stramenopiles *Poteriospumella,* whereas the genera that were more abundant in the lower stream included Amorphea *Nuclearia* and Stramenopiles *Paraphysomona* (Table S4).

### 9.3. ITS Amplicon Analysis

The ITS sequences from only one site (TD1) were successfully amplified and sequenced. Results from taxonomic classification showed that the five most abundant phyla were Ascomycota (53.96%), Chytridiomycota (29.95%), Basidiomycota (9.22%), Rozellomycota (0.81%), and Glomeromycota (0.26%). The 10 genera with the highest relative abundance were *Avachytrium* (18.57%), *Entophlyctis* (10.95%), *Cladosporium* (3.61%), *Capnobotryella* (2.54%), *Hannaella* (2.40%), *Glutinoglossum* (2.33%), *Sporobolomyces* (2.29%), *Inocybe* (0.96%), *Cryptococcus* (0.61%), and *Acremonium* (0.38%) (Table S5).

## 10. Diversity Estimation

### 10.1. Alpha Diversity

Alpha-diversity indices revealed the diversity, evenness, and richness within each study site. Shannon’s index indicates the diversity within site, in which richness, evenness, and rare OTUs played an essential role in the measure. Simpson’s index indicates diversity as Shannon’s index does, however, the rare OTUs play a minor role in the estimation. Chao1 and the number of identifiable OTUs reflect the richness within site.

**Figure 5:**
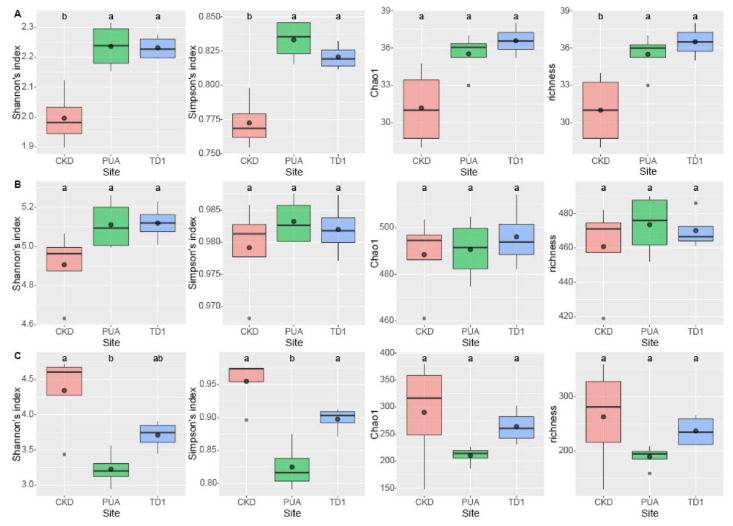
Boxplots of Alpha-diversity indices included Shannon’s index, Simpson’s index, Chao1, and richness (number of identifiable taxa) of *Cladophora* microbiomes collected from Nan River, Thailand. (**A**) Bacterial phyla. (**B**) Bacterial genera. (**C**) Eukaryotic genera. Different lowercase letters indicated statistically significant differences using Tukey’s HSD at a significant level of *p* < 0.05.

For the bacterial taxa, at the phylum level (Figure 5A), Shannon’s indices showed that PUA (2.24) and TD1 (2.23) were statistically more diverse than CKD (2.00) as *p* < 0.001. Simpson’s indices of PUA (0.83) and TD1 (0.82) were also statistically more diverse than CKD (0.77) as *p* < 0.005. Chao1 of CKD, PUA, and TD1 were 31.19, 35.53, and 36.58, where only the Chao1 of TD1 was statistically higher than CKD (*p* < 0.01). The richness or number of identifiable bacterial phyla of CKD, PUA, and TD1 were 31.00, 35.50, and 36.50, respectively, where the richness values of PUA and TD1 were statistically higher than that of CKD (*p* < 0.01).

At the genus level of the bacterial taxa (Figure 5–B), Shannon’s indices of CKD, PUA, and TD1 were 4.91, 5.11, and 5.12, which were not statistically different. Simpson’s index of the three sites was 0.98. Choa1 of CKD, PUA, and TD1 were 488, 491, and 496, respectively, which were not statistically different. The richness values of CKD, PUA, and TD1 were 461, 474, and 470, respectively, which also were not significantly different.

For eukaryotes, at the genus level (Figure 5C), Shannon’s index of CKD, PUA, and TD1 were 4.34, 3.23, and 3.71, where the diversity of CKD was statistically more diverse than PUA (*p* < 0.01). Simpson’s index of CKD (0.96) and TD1 (0.90) were statistically more diverse than PUA (0.8) at *p* < 0.001. Choa1 of CKD, PUA, and TD1 were 290, 210, and 264, which were not significantly different. The richness of CKD, PUA, and TD1 were 263, 190, 237, which were also not significantly different.

### 10.2. Beta Diversity

**Figure 6:**
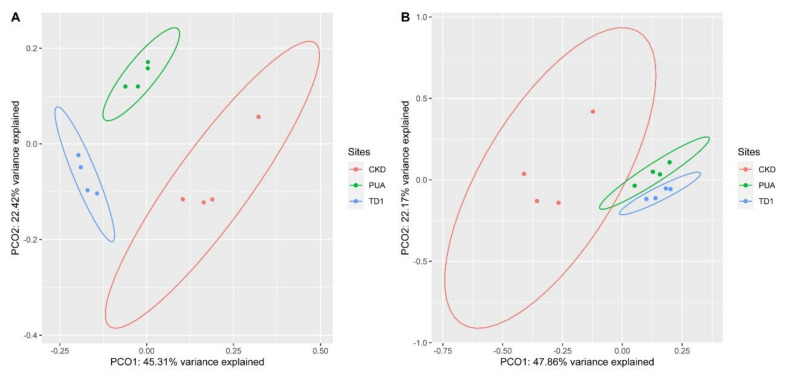
Principal Coordinate Analysis (PCoA) based on Bray–Curtis dissimilarity of *Cladophora* microbiomes collected from Nan River, Thailand. (**A**) Bacterial genera. (**B**) Eukaryotic genera.

Comparative analysis showed that the Bray-Curtis dissimilarity values of the bacterial phyla were not different among sites while the bacterial genera from the three study sites were significantly different from each other. The R value, which signifies dissimilarity between groups based on the Bray–Curtis dissimilarity values, resulted from both ANOSIM and permANOVA was 0.852 (*p* < 0.001). PCoA based on Bray–Curtis dissimilarity showed that the variance explained of PCo1 and PCo2 were 45.31% and 22.42% (67.73% in total), where the PCoA plot showed the prominent cluster of each site, representing the similarity of bacterial genera within each site (Figure 6A).

For eukaryotes, at the genus level, the R value obtained from both ANOSIM and permANOVA was 0.630 (*p* < 0.001). Results from PCoA based on Bray–Curtis dissimilarity showed that the variance explained of PCo1 and PCo2 were 47.86% and 22.17% (70.03% in total), where the PCoA plot showed a cluster of PUA and TD1 that distinguished these sites from CKD (Figure 6B).

## 11. Common Members of Freshwater *Cladophora* Microbiomes

The wide distribution range of the freshwater *Cladophora* sp. made it interesting to investigate whether the alga present in different geographical areas and different ecosystems (lotic and lentic) host a common group of microbes. Therefore, we compared the microbiomes of *Cladophora* found in this study to those of previously reported studies. Results showed that among the reports of *Cladophora* microbiomes, the *Cladophora* microbiome shared more common taxa with results reported from Lake Mendota, USA [6,9] than to Lake Michigan [7]. Interestingly, though different in abundance, 17 bacterial genera were present in microbiomes of all these freshwater *Cladophora* studies. These included Bacteroidetes *Chryseobacterium, Flavobacterium, Terrimonas,* Deinococcus-Thermus *Deinococcus*, Proteobacteria *Acidovorax, Acinetobacter, Altererythrobacter, Aquabacterium, Bdellovibrio, Hydrogenophaga, Hyphomicrobium, Methylotenera, Novosphingobium, Pseudomonas, Pseudorhodobacter, Rhodobacter,* and *Sphingopyxis* (Table S6).

## 12. Discussion

Along the river basin, *Cladophora* appeared as tufts/thalli attached on the rock surface across the river streams where the water level was about knee height. Among the collecting sites, *Cladophora* dominated the upper regions of the Nan River (CKD, PUA, and TD1), where the water temperatures were lower and the main substrates were rocks and pebbles.

Presence of similar algal morphology and identical sequences of selected gene markers suggested that the algal samples collected in this study belong to the same algal population. The best 100 BLASTN results of *Cladophora* amplicons in this study were sequences from published and unpublished *Cladophora* species collected from different countries, showing the same total score, percent identity, and E-value (Table S2). This suggested that the amplified region had been conserved across different *Cladophora* populations. We, therefore, could not annotate the algal sample further than the genus level.

It was interesting to us that although we found that our amplicons were identical to sequences reported from other locales, these amplicons were not identical to any of the reported freshwater *Cladophora* found in Thailand or the neighboring counties, e.g., GenBank accession LT607372.1 [54] and accessions JQ071987.1-JQ072004.1 [55]. This suggests that there might be more than one population of the freshwater *Cladophora* endemic to the region, which has not yet been explored.

### Microbiomes of Lentic Cladophora sp.

The nature of *Cladophora*, producing high-surface-area filaments attached to substrates in the river basin, makes it crucial in the ecosystem by providing microhabitats for other microbial organisms. Results from amplicon analysis revealed that the dominant epiphytic eukaryotes in the three sampling sites were Amorphea and Stramenopiles, which agreed with results from light microscopy, where we observed zooplankton and photosynthetic stramenopiles *Cocconeis*, *Gomphonema*, *Synura*, *Synedra,* and *Navicula* on the *Cladophora* cell surface.

Among the diatoms, *Cocconeis* and *Gomphonema* were present in all the sampling replicates, judged by both microscopic and molecular evidence, where the relative abundance of *Cocconeis* ranged from 4.9% at CKD to 27.4% at PUA, and the relative abundance of *Gomphonema* ranged from 4.6% at TD1 to 0.8% at PUA. The presence of dominant *Cocconeis* and *Gomphonema* on the filament surface was similar to results from previous studies [9,10], which hypothesized that this diatom’s cell shape allowed it to avoid grazing and maintain firm attachment to the algal filament.

In addition to the dominant diatom *Cocconeis*, a few other diatoms were also present in all the sampling sites, e.g., *Achnanthidium*, *Amphora*, *Craticula*, *Cymbella*, *Epithemia*, *Fistulifera*, *Gomphonema*, *Melosira*, *Navicula*, *Nitzschia*, *Pinnularia*, *Planothidium*, *Rhopalodia*, and *Thalassiosira*. These diverse diatoms on the algal filaments might be explained by ability to tolerate shading that occurs in the *Cladophora* tuft. Within the algal tuft, the high density of algal filaments results in variation in light intensity. By having such ability, the diatoms can maintain their abundance by upregulating the expression of genes involved in transcription and photosynthesis to compensate for the lower light intensity [56].

Presence of these diatoms on the *Cladophora* surface promotes high diversity of the algal microbiome as these diatoms release their photosynthetic exudates or exopolysaccharides, which provide the building blocks of biofilms that encase the algal filaments, e.g., [57,58]. According to results from our amplicon analysis, there were 698 unique bacterial genera and 575 unique eukaryotic genera present in the *Cladophora* microbiome (Table S3 and Table S4).

Alpha indices suggested that the diversity of bacterial phyla at PUA and TDI was higher than that of CKD, whereas the eukaryotic genera were more diverse at CKD than at PUA and TD1. In addition, clusters obtained from the PCoA plots suggested that the diversity of the eukaryotic genera and the bacterial genera was more similar within site than among sites. It is unclear at this stage why the diversity of these microbiomes was statistically different. However, we believe that it is not mainly affected by the trophic states of the water at the collecting site as the trophic states do not correlate with the diversity level of the organisms [59,60,61]. In this study, although we failed to collect the physical properties of the water from the sampling sites, secondary data obtained from the annual report of the local environmental organization showed that the trophic state of the water in the Nan River fluctuated. The values of water quality index, dissolved oxygen, biological oxygen demand, and ammonia nitrogen content appeared to fluctuate along the course of the river (Figure S2). To understand this better, an investigation of the biomes presents at the riverbank or the adjacent floodplains might be needed. Variation in effluent inputs to the river might also be important.

Although our results showed different bacterial diversity along the river course, we found a total of 227 identifiable bacterial genera that were commonly present in all sites that might be involved in various important ecological functions (Table 1). Among these common genera, some appeared to be more abundant when compared to other common genera across the three study sites. These included taxa involved in processes like cellulose degradation (*Bacteroides* and *Paludibacter*), cobalamin (vitamin B_12_) biosynthesis (*Flavobacterium*), fermentative hydrogen production (*Acetobacteroides* and *Clostridium sensu stricto 12*), ammonium oxidation (*Pirellula*), amino acid fermentation (*Acidaminobacter*), dissimilatory reduction of nitrate to ammonium (*Pelosinus, Aeromonas*, and *Lacunisphaera*), nitrite reduction (*Dechloromonas*), nitrate reduction (*Vogesella*), sulfur reduction (*Fusibacter*), polyphosphate accumulation (*Acinetobacter* and *Propionivibrio*), denitrifying phosphorus-accumulation (*Dechloromonas*), degradation of aromatic compounds (*Hydrogenophaga*), anaerobic chemoheterotroph (*Fimbriiglobus*), and bacteria with no known specific ecological function, e.g., *Emticicia*, *Pseudarcobacter,* and WCHB1-32.

The majority of the fungi present within the samples collected from TD1 were fungi that function in decomposition, e.g., *Avachytrium*, *Entophlyctis*, *Glutinoglossum*. Other fungi were those known to be involved in (1) parasitism in algae, amoebae, and other fungi, (2) plant-fungal interaction, (3) predation, and even (4) mutualism as in the lichen-forming fungi (Table 2). We believe that, while some fungi were present temporarily (e.g., lichen-forming fungus, phylloplane fungi, and plant pathogens), a portion of these fungi live within the host algal microbiome. However, to know which fungi live in the associations and which fungi are there temporarily due to the influx from the nearby flood plain, more replications of *Cladophora* microbiomes from more study sites are needed.

## 13. Common Members of Freshwater *Cladophora* Microbiomes

We re-analyzed the published data in archives [6,7,9] and investigated if there are shared taxon between the lotic and lentic freshwater *Cladophora*. As expected, only a few taxa were commonly present in all algal microbiomes as the collecting sites were located in different hemispheres and hydrological systems. All the common taxa were bacteria, which were known to be involved in important ecological activities, including cobalamin (vitamin B_12_) biosynthesis (*Flavobacterium* and *Pseudomonas*), extracellular polymeric substance secretion (*Terrimonas*), aerobic chemoorganotrophy (*Chryseobacterium* and *Novosphingobium*), anoxygenic phototrophy (*Rhodobacter*), polyphosphate accumulation (*Acinetobacter*), denitrification (*Acidovorax* and *Methylotenera*), degradation of alkane (*Aquabacterium* and *Hyphomicrobium*), and aromatic compounds (*Hydrogenophaga*). The presence of these common bacteria might be due to their crucial roles for *Cladophora* dispersal and survival or the wide distribution of the bacteria themselves. To answer the question, a further investigation of their interactions is needed.

**Figure 7:**
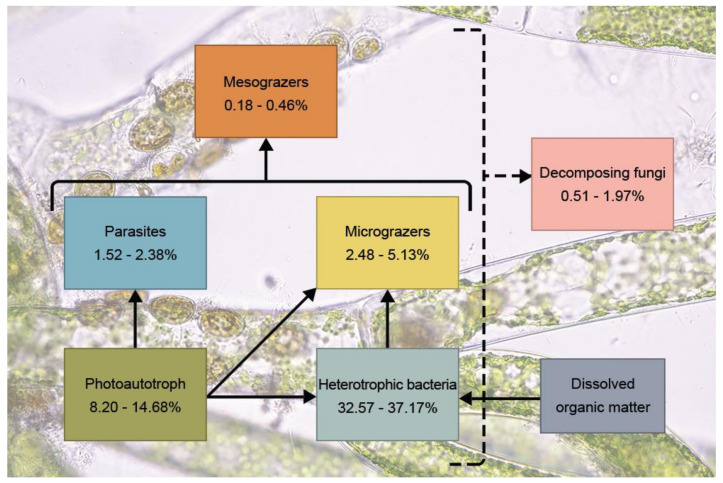
Average of the abundance of organisms living in the *Cladophora* microbiomes within each collecting site. For the identifiable taxa, the microbiomes were dominated by the heterotrophic bacteria, followed by photoautotrophs, micrograzers, parasites, decomposing fungi, and mesograzers.

It is challenging to infer interactions between organisms living in the lotic environment as there is an influx and efflux of nutrients occurring continuously. This turbulence affects the richness and abundance of the organisms living in the algal microbiomes, e.g., [236,237,238]. Even so, data from this study revealed that *Cladophora* microbiomes were dominated by heterotrophic bacteria (32.57–37.17%), followed by photoautotrophs (8.20–14.68%), micrograzers (2.48–5.13%), parasites (1.52–2.38%), decomposing fungi (0.51–1.97%), and mesograzers (0.18–0.46%), as shown in Figure 7. The presence of similar relative abundances of organisms belonging to different trophic levels and decomposers suggested that there might be some unique interactions or activities present in the lotic freshwater *Cladophora* microbiomes that have not yet been investigated.

In summary, we have shown that the *Cladophora* sp. that we sampled at three sites along the Nan River belongs to the same algal species. Our amplicon analyses revealed that this alga harbored diverse groups of microorganisms whose taxonomic richness and abundance varied among the sampled sites. About 50 percent of the identifiable taxa were shared among the *Cladophora* microbiomes. These organisms span different trophic levels in the food chain and putatively serve various ecological services.

## Figures and Tables

**Table 1:** Putative ecological functions inferred for bacterial genera commonly present in the *Cladophora* microbiomes collected from CKD, PUA, and TD1.

Ecological Functions	Bacterial Genera
Nitrogen cycling	
Denitrification	*Acidovorax* [62] *Arenimonas* [63] *Chromobacterium* [64] CL500-29 marine group [65] *Comamonas* [66] *Devosia* [67] *Hyphomicrobium* [68] *Leptothrix* [63] *Methylotenera* [69] *OLB13* [70] *Opitutus* [67] *Paracoccus* [63] *Pseudomonas* [67] *Rhodobacter* [67] SM1A02 [71] *Thauera* [72]
Denitrifying phosphorus-accumulation	*Candidatus* Accumulibacter [73] *Dechloromonas* [73]
Dissimilatory reduction of nitrate to ammonium	*Aeromonas* [74] *Geobacter* [74] *Lacunisphaera* [74] *Pelosinus* [75]
Ammonium oxidation	*Blastopirellula* [76] *Brevifollis* [77] Ellin6067 [78] *Gemmata* [79] mle1-7 [78] oc32 [78] *Pirellula* [79]
Nitrate reduction	*Noviherbaspirillum* [80] *Rhizobacter* [81] *Sulfurospirillum* [82] *Thauera* [82] *Vogesella* [83]
Nitrite oxidation	*Candidatus* Nitrotoga [84]
Nitrite reduction	*Arenimonas* [73] *Azoarcus* [73] *Dechloromonas* [85] *Haliangium* [85] *Rhodoferax* [86] *Sulfuritalea* [85]
Nitrogen fixation	*Anaeromyxobacter* [87] *Azospira* [88] *Dechloromonas* [89] *Devosia* [90] *Mesorhizobium* [91] *Methylocystis* [92] *Nordella* [93] *Pelomonas* [94] *Rhizobium* [95] *Shinella* [96]
Nitrous oxide reduction	*Gemmatimonas* [97]
Iron cycling	
Fe (II) oxidation	*Curvibacter* [98] *Leptothrix* [99] *Noviherbaspirillum* [80] *Sideroxydans* [100] *Undibacterium* [98]
Fe (III) reduction	*Acidibacter* [101] *Geobacter* [99] *Rhodoferax* [99]
Manganese cycling	
Mn (II) oxidation	*Pedomicrobium* [102] *Sideroxydans* [100]
Mn (IV) reduction	*Geobacter* [99] *Rhodoferax* [99]
Phosphorous cycling	
Polyphosphate accumulation	*Acinetobacter* [103] *Arcicella* [104] *Limnohabitans* [104] *Propionivibrio* [105]
Sulfur cycling	
Sulfate reduction	*Desulfobulbus* [106] *Desulfomicrobium* [107] *Desulfovibrio* [108]
Sulfur oxidation	*Limnobacter* [109] *Meiothermus* [110]
Sulfur reduction	*Fusibacter* [111]
Uranium cycling	
U (IV) reduction	*Anaeromyxobacter* [112] *Geobacter* [112]
Degradation	
Alkane degradation	*Aquabacterium* [113] *Tropicimonas* [114]
Cellulose degradation	*Aquitalea* [115] *Bacteroides* [116] *Caulobacter* [117] *Cellvibrio* [118] *Cloacibacterium* [119] *Cytophaga* [120] *Exiguobacterium* [119] *Ilumatobacter* [121] *Paludibacter* [119] *Roseimarinus* [122] *Ruminiclostridium* [123]
Chitin degradation	*Chitinibacter* [124] *Chitinimonas* [125] *Massilia* [126] SH-PL14 [127]
Degradation of aromatic compounds	*Acinetobacter* [128] *Azoarcus* [99] *Hydrogenophaga* [129] *Hyphomicrobium* [130] *Leptothrix* [99] *Limnobacter* [128] *Methylibium* [131] *Ottowia* [130] *Pseudomonas* [128] *Sulfuritalea* [130] *Thauera* [132]
Degradation of biodegradable plastics	*Sphingopyxis* [133]
Volatile fatty acid degradation	*Ohtaekwangia* [134]
Other organic pollutant degradation	*Rheinheimera* [135]
Vitamin biosynthesis	
Cobalamin (vitamin B_12_) biosynthesis	*Bacillus* [136] *Candidatus* Udaeobacter [137] *Flavobacterium* [138] *Mycobacterium* [70] *Porphyrobacter* [139] *Porphyromonas* [140] *Pseudomonas* [141]
Phototrophy	
Cyanobacterial phototrophy	*Chamaesiphon* [142] *Cyanobium* [143]
Bacterial phototrophy	*Chloroflexus* [144]
Anoxygenic phototrophy	NOR5/OM60 clade [145] *Rhodobacter* [146] *Rhodoferax* [147] *Tabrizicola* [148]
Photoheterotrophy	*Rubrivivax* [149]
Chemotrophy	
Aerobic chemoheterotrophy	*Armatimonas* [150] *Fimbriiglobus* [151] *Flavisolibacter* [152] *Hirschia* [153] *Lewinella* [154] *Phaeodactylibacter* [155]
Aerobic chemoorganotrophy	*Ahniella* [156] *Albidovulum* [157] *Bryobacter* [158] *Chryseobacterium* [159] *Haloferula* [160] *Hyphomonas* [161] *Ideonella* [162] *Larkinella* [163] *Lysobacter* [164] *Novosphingobium* [165] *Polaromonas* [166] *Runella* [167] *Stenotrophobacter* [168] *Truepera* [169] *Zavarzinella* [170]
Anaerobic chemoorganotrophy	*Anaeromusa and Anaeroarcus* [171] *Phascolarctobacterium* [172] *Saccharofermentans* [173] *Sporomusa* [174]
Carbohydrate fermentation	*Alistipes* [175] *Ferruginibacter* [176] *Prevotella* 9 [177] *Treponema* [178] *Vallitalea* [179]
Animo acid fermentation	*Anaerovorax* [180] *Acidaminobacter* [181]
Fermentative hydrogen production	*Acetobacteroides* [182] *Clostridium sensu stricto 1* [183] *Clostridium sensu stricto 12* [184] *Cytophaga xylanolytica* [185]
Other	
Antifungal effect	*Duganella* [186]
Predator	*Bdellovibrio* [187] *Herpetosiphon* [188]
Methane oxidation	*Methylocystis* [92] *Methylovulum* [189]
Methylotrophy	*Gemmobacter* [190] OM43 clade [191]
Extracellular polymeric substance (EPS) secretion	*Terrimonas* [192]
Biosorption of heavy metals	*Sphaerotilus* [193]

**Table 2:** Fungal genera commonly present in the *Cladophora* microbiomes and their putative ecological functions.

Ecological Functions	Fungal Genera
Decomposing fungi	
Saprotrophy	*Acremonium*^a^ [194] *Alternaria* ^a^ [195] *Arthrinium* [196] *Aspergillus* ^a^ [197] *Avachytrium* ^b^ [198] *Capnobotryella* ^b^ [199] *Chaetospermum* [200] *Chytriomyces* [201] *Cladosporium* ^a^ [202] *Emericellopsis* [203] *Entophlyctis* ^b^ [204] *Fusarium* ^a^ [205] *Galactomyces* [206] *Gibellulopsis* ^a^ [207] *Glutinoglossum* ^b^ [208] *Hannaella* ^a^ [209] *Helicascus* [210] *Inocybe* ^b^ [211] *Lentithecium* [212] *Mortierella* ^a^ [213] *Nowakowskiella* [214] *Ochroconis* ^a^ [215] *Piromyces* [216] *Pyrenochaeta* ^a^ [217] *Sporobolomyces* ^a^ [218] *Wiesneriomyces* [219]
Parasitism	
Endoparasitic chytrid	*Rozella* [220]
Hyperparasites on other fungi	*Cladosporium*^a^ [202]
Parasites of algae	*Entophlyctis*^b^ [221] *Phlyctochytrium* [222] *Rhizophydium* [223]
Parasites of amoebae	*Acaulopage* [224] *Cochlonema* [224] *Paramicrosporidium* [220]
Parasitoids of algae	*Aphelidium* [225] *Paraphelidium* [226]
Plant-fungal interaction	
Ectomycorrhizal fungi	*Inocybe*^b^ [211]
Endophytic fungi	*Acremonium*^a^ [211] *Alternaria* ^a^ [195] *Arthrinium* [196] *Cladosporium* ^a^ [202]
Phylloplane fungi	*Cladosporium*^a^ [202] *Geotrichum* [227] *Hannaella* ^a^ [209] *Occultifur* ^a^ [228] *Pichia* [229] *Rhodotorula* ^a^ [230] *Sporobolomyces* ^a^ [229] *Vishniacozyma* ^a^ [230]
Plant growth-promoting fungi	*Mortierella*^a^ [231]
Plant pathogen	*Alternaria*^a^ [195] *Arthrinium* [196] *Cladosporium* ^a^ [202] *Fusarium* ^a^ [232] *Gibellulopsis* ^a^ [207] *Sporisorium* ^a^ [233]
Predation	
Amoebophagous fungi	*Stylopage* [234]
Nematophagous fungi	*Stylopage* [235]
Mutualism	
Lichen-forming fungi	*Capnobotryella*^b^ [198]

^a^ Found in both 18S rDNA and ITS amplicon analysis. ^b^ Found in ITS amplicon analysis only.

## Data Availability

Raw amplicon metagenomic sequences were deposited in the NCBI SRA as BioProject PRJNA761577, BioSample SAMN21356006 (CKD), SAMN21356007 (PUA), and SAMN21356008 (TD1).

## Supplementary Materials

The followings are available online at https://www.mdpi.com/article/10.3390/plants10112266/s1, Figure S1: Alignment of 18S rDNA amplicons collected from CKD, PUA, and TD1 showing identical sequences of Cladophora sp. use in this study. Figure S2: The water quality along the Nan River collected from February 2019 to December 2020 from locations 1–4, span the collecting time in this study in March 2020. (a) values of the water quality index, (b) dissolved oxygen, (c) biological oxygen demand, (d) ammonia nitrogen content, and (e) the locations of the water sampling stations. Table S1: Primer sequences and PCR conditions for amplification of 16S rDNA, 18S rDNA, and ITS amplicons. Table S2: Top 100 BLASTN hit of the Cladophora 18S rDNA amplicon. Table S3: Bacterial genera and their abundance found in each replicate of collecting cites CKD, PUA, and TD1. Table S4: Eukaryotic genera and their abundance found in each replicate of collecting cites CKD, PUA, and TD1. Table S5: Fungi phyla and their abundance found in TD1. Table S6: Common members of freshwater *Cladophora* microbiomes. Green boxes represent the presence of the corresponding taxa.
