# Supplementary figures and images for: Spatial Variation of Cladophora Epiphytes in the Nan River, Thailand
Source: Plants (Basel). 2021 Oct 22;10(11):2266. doi: 10.3390/plants10112266 (PMC8622721; doi:10.3390/plants10112266)

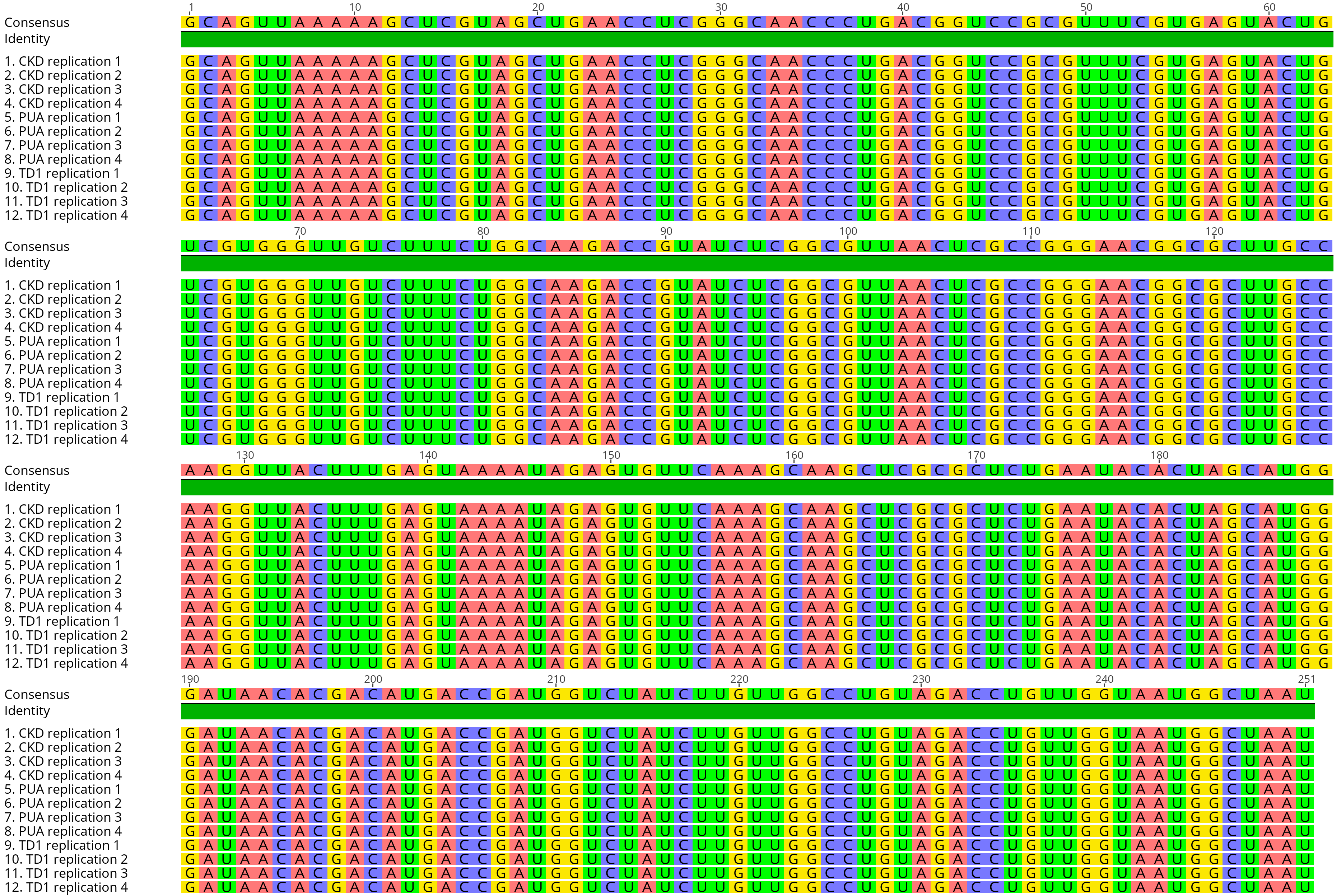

Supplement: Supplementary file 1 [file plants-10-02266-s001.zip › FIGURE S1.png]

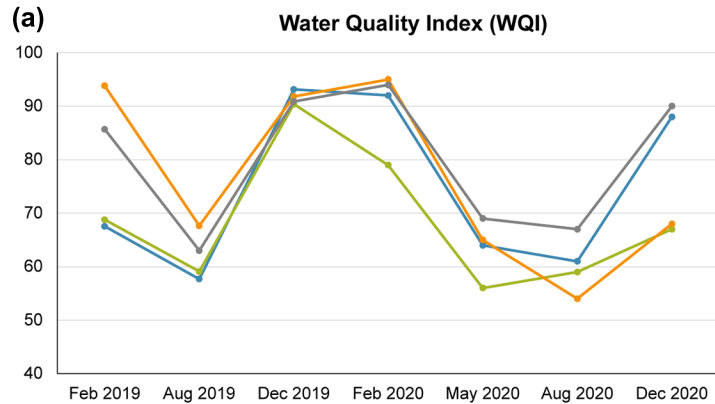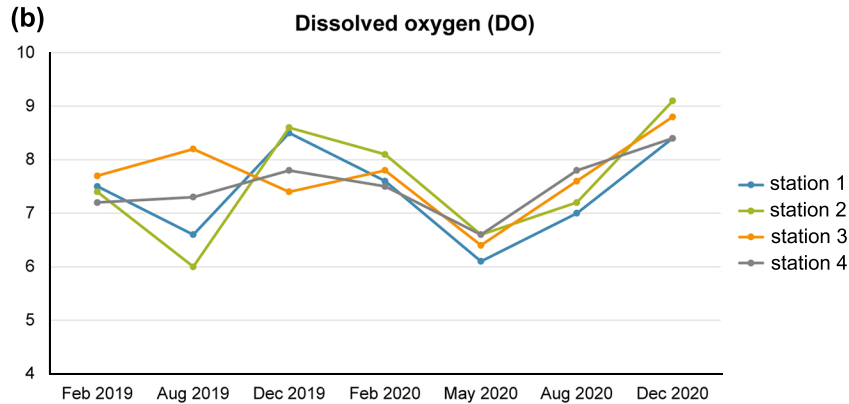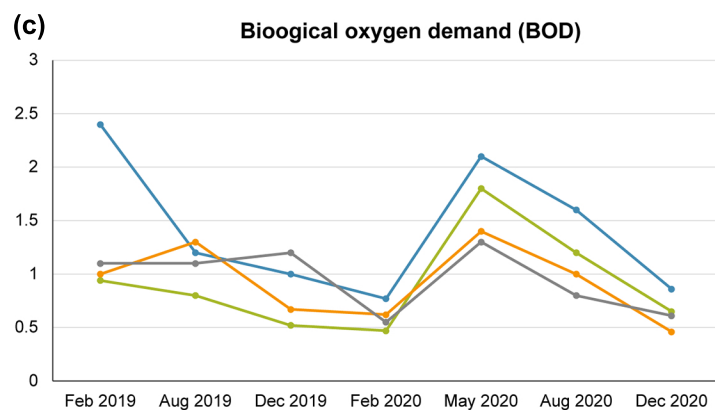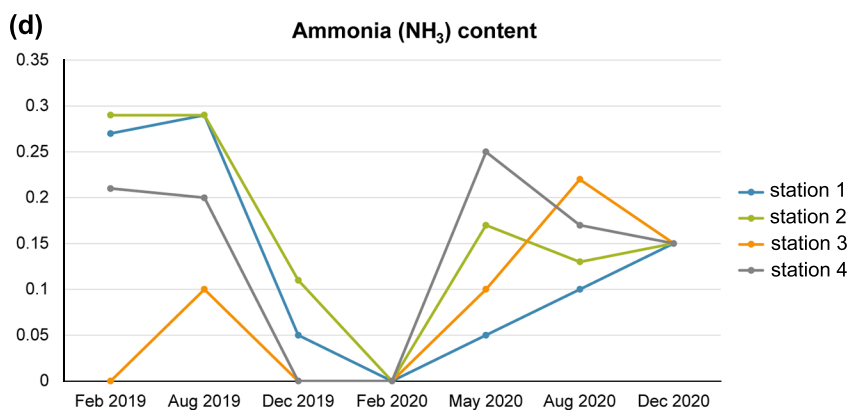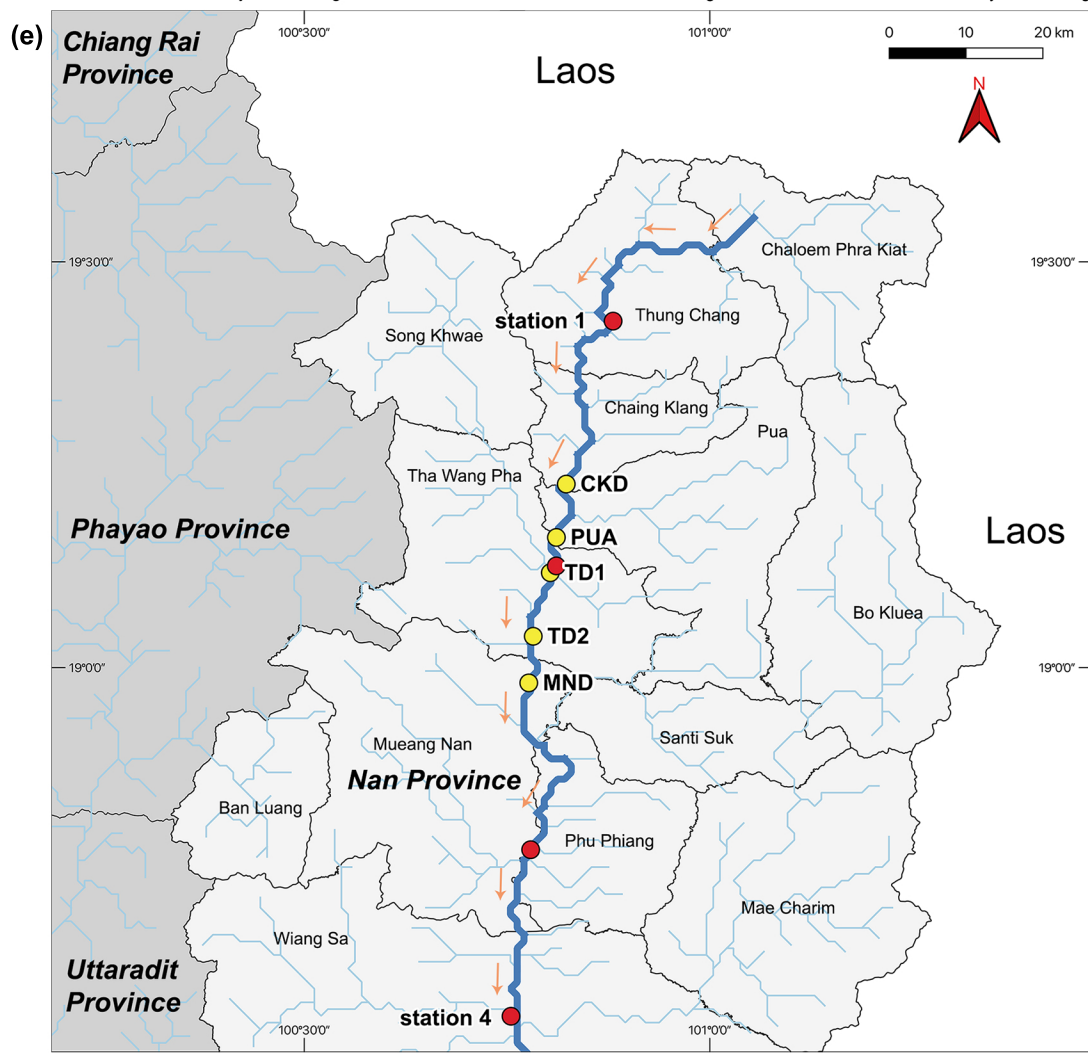

Supplement: Supplementary file 1 [file plants-10-02266-s001.zip › FIGURE S2_revised.pdf]
